# In Situ Investigation of Dynamic Silver Crystallization Driven by Chemical Reaction and Diffusion

**DOI:** 10.34133/2020/4370817

**Published:** 2020-02-10

**Authors:** Ting Liu, Xiangyu Dou, Yonghui Xu, Yongjun Chen, Yongsheng Han

**Affiliations:** ^1^State Key Laboratory of Multiphase Complex Systems, Institute of Process Engineering, Chinese Academy of Sciences, 100190 Beijing, China; ^2^State Key Laboratory of Marine Resource Utilization in South China Sea, Hainan University, 570228 Haikou, China; ^3^School of Chemical Engineering, University of Chinese Academy of Sciences, 100049 Beijing, China

## Abstract

Rational synthesis of materials is a long-term challenging issue due to the poor understanding on the formation mechanism of material structure and the limited capability in controlling nanoscale crystallization. The emergent in situ electron microscope provides an insight to this issue. By employing an in situ scanning electron microscope, silver crystallization is investigated in real time, in which a reversible crystallization is observed. To disclose this reversible crystallization, the radicals generated by the irradiation of electron beam are calculated. It is found that the concentrations of radicals are spatiotemporally variable in the liquid cell due to the diffusion and reaction of radicals. The fluctuation of the reductive hydrated electrons and the oxidative hydroxyl radicals in the cell leads to the alternative dominance of the reduction and oxidation reactions. The reduction leads to the growth of silver crystals while the oxidation leads to their dissolution, which results in the reversible silver crystallization. A regulation of radical distribution by electron dose rates leads to the formation of diverse silver structures, confirming the dominant role of local chemical concentration in the structure evolution of materials.

## 1. Introduction

It is a long-term research interest of scientists and engineers to synthesize material structures rationally due to different structured materials having different properties and applications [1, 2]. Great efforts have been devoted to disclose the formation mechanism of material structures and to develop routes for the rational synthesis [3–5]. Since the formation of materials occurs at the atomic scale at the time down to nanoseconds, it is difficult to directly observe the formation of diverse structures. This difficulty challenges our knowledge to understand the crystallization process as well as to formulate rational routes. The trial-and-error method is still the main solution to develop advanced materials, which leads to a high cost of time and risk. In 2011, the US government initiated the Materials Genome Initiative to speed up material discoveries and development by integrating experiments, computation, and theory, which aims at deploying advanced materials twice as fast, at a fraction of the cost by conducting vast experiments and computation and sharing diverse datasets [6]. At the same time, another research line is kept running to open the black box by employing in situ instruments and new methodology [7, 8].

Electrospray ionization mass spectrometry and nuclear magnetic resonance spectroscopy were employed to monitor the reaction intermediates in the evolution of thiolate-stabilized silver nanoclusters, which not only reveals the formation mechanism of nanoclusters but also supports the total synthesis routes and mechanisms for atomically precise metal nanoclusters [9–12]. To see the crystallization process, optical microscopy was used to track nucleation processes [13], but it cannot resolve structures below a few hundred nanometers in diameter without fluorescent labeling. X-ray scattering and spectroscopic techniques could detect nuclei at early times but only capture the ensemble evolution of the system [14]. In situ scanning probe microscopy has been successfully applied to the nucleation and growth in many surface-mediated systems due to its ability to image surfaces with high spatial resolution [15, 16]. The surface scanning technique has limitations to observe the crystallization process in a solution. Upon this demand, the liquid phase electron microscope was developed, which combines the temporal and spatial resolution of electron microscopy with the ability to image events occurring in bulk solutions [17, 18]. With the use of the liquid phase electron microscope, the crystallization of barium tungstate was monitored, which indicated that the complex morphologies grow through a classical growth mechanism, rather than the long-term supposing nonclassical crystallization [19]. The in situ electron microscope is a powerful tool to disclose the formation of materials [20, 21]. It provides insights, not readily accessible by other means, into the phenomena of the nucleation and growth of nanoparticles [22], assembly of nanoparticles [23], electrochemical processes [24, 25], etc. The direct observations of crystallization have greatly increased our understanding on the growth mechanisms of materials.

To see the formation of materials is to disclose the formation mechanism of structures. Another important issue is to figure out the secret hands which have the ability to put each atom in a position. In 1953, Alan Turing, the pioneer of artificial intelligence and the theory of computation, proposed that most patterns of biology may be determined by the diffusion and reaction of morphogens [26]. Although the morphogens have not been discovered, the principle of reaction and diffusion mechanism was confirmed in many systems recently [27, 28]. We took the diffusion and reaction of chemicals as two hands to disclose their role in the formation of material structures [29–32]. By regulating chemical diffusion and reaction, we synthesized diverse morphologies of silver particles [29, 30], as well as other materials [31, 32], confirming the general role of chemical diffusion and reaction in shaping materials. At different diffusion and reaction conditions, the chemical distribution around the growth front of crystals is different, which results in a kinetics-dominated anisotropic growth of structures. Owing to the continuous diffusion and reaction in the growth of materials, the chemical distribution changes spatiotemporally in the reactor. How the ever-changing chemical distribution influences the growth of material structure as well as the final shape of products is an unclear issue. If the formation process could be monitored online and correlated with the change of chemical distribution in real time, the dependence of structure evolution on the chemical distribution is expected to be clarified, which may benefit the design and rational synthesis of material structures.

In this study, an in situ scanning electron microscope (SEM) is employed to record silver crystallization followed by associating the structure evolution with the chemical distribution in a liquid cell loaded in the SEM. The liquid cell confines a layer of the solution of silver nitrate between two membranes and is hermetically sealed for the high vacuum of the microscope. The electron beam enters into the cell through an electron-transparent silicon nitride film with a thickness of 100 nanometers, enabling a real-time imaging of silver crystallization in the liquid. The electron beam firstly transfers energy to the irradiated medium, which results in the generation of radical and molecular species [33]. The irradiation products continue to react, causing a cascade of chemical reactions and a production of various additional species [34]. Owing to the diffusion and reaction of these species, the chemical distribution in the reactor changes spatiotemporally. At a certain electron dose rate, the fluctuation of reductive and oxidative radicals leads to the alternative dominance of reduction and oxidation reaction, resulting in a dynamic silver crystallization. A regulation of the electron dose rate changes the radical concentration, which leads to the formation of diverse structures of crystals, confirming the dominant role of chemical concentration in the structural evolution of crystals.

## 2. Results

### 2.1. Spatiotemporal Distribution of Radicals in the Liquid Cell

**Figure 1:**
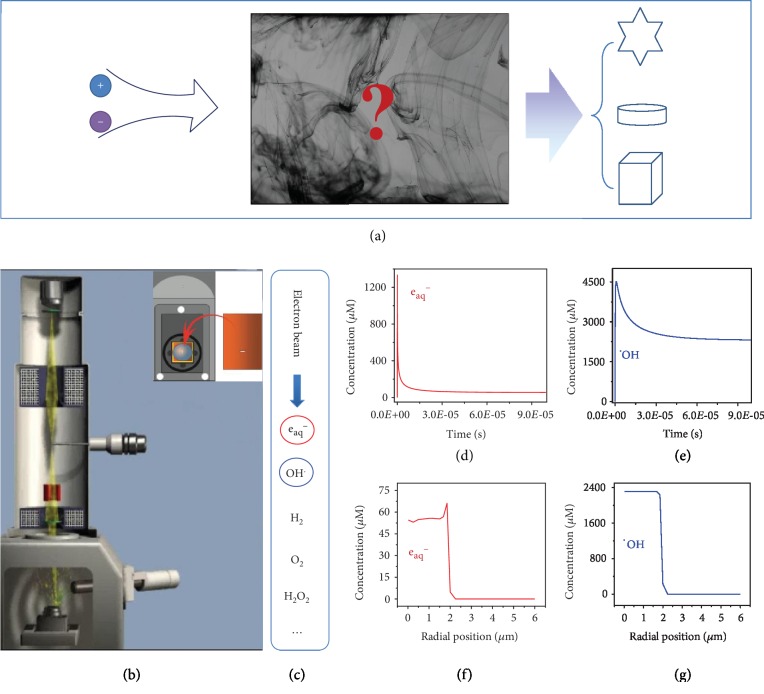
Radical distribution in a liquid cell. (a) An illustration of the unclear relationship between the reaction conditions and the structures of final products, in which chemical distributions may play a bridging role. (b) An illustration of the in situ electron microscope, in which a sealed cell (inset) containing silver nitrate solution is loaded into an electron microscope.(c) The radical products generated by the irradiation of an electron beam to water. The concentrations of hydrated electron and hydroxyl radicals changing with time (d, e) and space (f, g). (d) and (e) show a quick generation of these two products followed by a quick vanishing as a result of reactions. (f) and (g) show a steady state concentration of these two products in the region irradiated and a sharp reduction outside the irradiated region.

In the synthesis of materials, the chemical distribution in the reactor changes with time and space. How this spatiotemporal change of chemical distribution influences the structure evolution of materials is unclear, as illustrated in Figure 1(a), which is the main issue to be discovered in this paper. To monitor the formation of materials structure, an in situ scanning electron microscope (SEM) is employed, in which a sealed cell containing silver nitrate solution is loaded in a SEM, as shown in Figure 1(b). The electron beam enters into the cell through glass windows. The beam irradiates water molecules forming a variety of irradiated products, including hydrated electrons, hydroxyl radicals, and hydrogen, as shown in Figure 1(c). The hydrated electron is a strong reducing agent with a reduction potential of -2.9 V while the hydroxyl radicals is the oxidative agents with a standard potential of 2.7 V [35, 36]. They are the main redox components in the cell and induce complex reactions. The concentration of these two radicals is calculated by setting up a kinetic model in which the chemical reaction and diffusion are considered. The concentration of hydrated electrons increases rapidly in a short time and then decreases to a pretty low steady state concentration, as shown in Figure 1(d), which indicates that both the generation and the vanishing of hydrated electron are very fast. The disappearance of hydrate electrons may be resulted from their reaction with oxidative substances, such as oxygen and hydrogen peroxide generated by the irradiation. The reaction with hydroxyl radicals also cannot be excluded. The concentration of hydroxyl radicals changes similarly as that of the hydrated electron. An increase of concentration is followed by a decrease and reaches a steady state concentration finally, as shown in Figure 1(e). However, its steady state concentration is much higher than that of the hydrated electron.

The chemical distribution in different locations is calculated by solving the diffusion-reaction equation via ANSYS Fluent software. 79 chemical reactions were considered in the system [37]. In the irradiation region (*r*_*p*_ < 2 *μ*m), the electron dose rate is set to 7.33 × 10^11^ Gy/s while outside the irradiation region (*r*_*p*_ > 2 *μ*m), the electron dose rate is zero. The concentration of the main products of the hydrated electron and hydroxyl radical changes with the location, as shown in Figures 1(f) and 1(g). In the irradiation region, the concentration of the hydrated electron is kept in the range of 50 to 60 *μ*M. There is a peak at the boundary of the irradiation region followed by a fast decrease to zero outside the region, as shown in Figure 1(f). This peak is attributed to the diffusion of oxidative radicals from the boundary to the bulk. This diffusion leads to a reduction of the oxidative radicals at the boundary, which results in a decrease of the redox reaction at the boundary. The low steady state concentration of the hydrated electron in the irradiation region makes the peak distinct. The concentration of the hydroxyl radical is kept at the range of 2250 to 2500 *μ*M in the irradiation region followed by a fast decrease to zero outside the region, as shown in Figure 1(g). The high concentration of hydroxyl radicals makes the change at the boundary invisible and there is no peak at the boundary. The above results show that the hydrated electron and hydroxyl radical are mainly present in the irradiation region.

**Figure 2:**
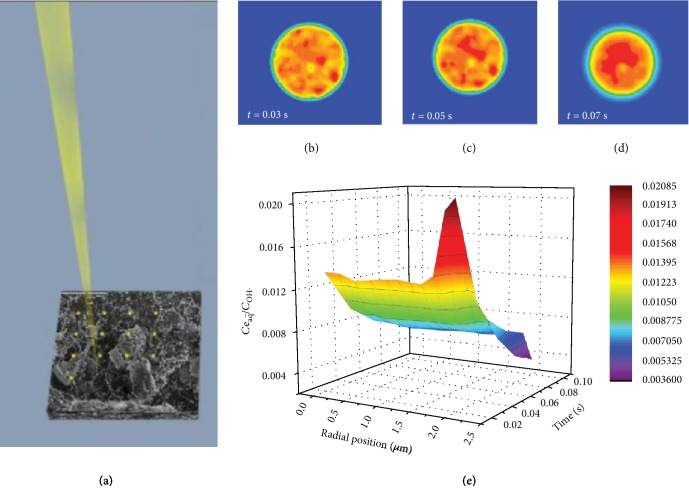
The distribution of hydrated electron in a selected region. (a) An illustration of the point-by-point scanning mode of a scanning electron microscope. (b–d) The hydrated electron distribution in the selected region at different times. (e) The ratio of the concentrations of hydrated electron and hydroxyl radicals in the region varies with time and location.

A scanning electron microscope scans the surface of samples via a point-by-point mode, as illustrated in Figure 2(a). Figure 2(b) shows the distribution of the hydrate electron in a selected region with a diameter of 2 *μ*m at the irradiation time of 0.03 s. The selected region contains many electron scanning points which have a diameter of approximately 5 nm upon the voltage applied. The point-by-point scanning in the selected area followed by radical reaction and diffusion causes a location-dependent distribution of hydrate electrons. With the advance of the irradiation, the concentration of hydrated electron changes, as shown in Figures 2(c) and 2(d). To characterize the redox ability of the irradiated products, the ratio (*J*) of the concentrations of hydrated electrons and hydroxyl radicals is calculated at the dose rate of 7.33 × 10^11^ Gy/s. To have a clear view of the distribution of these redox radicals in the liquid cell, a three-dimensional diagram of the *J* value at a function of time and space is plotted, as shown in Figure 2(e). The red peaks and blue valleys are observed in the irradiation region. The red peaks indicate a relatively high concentration of hydrated electrons while the blue valley indicates a high concentration of hydroxyl radicals. If the point-by-point scanning mode is considered, a fluctuation of the *J* value in the whole range could be expected. How the fluctuation influences the structure evolution of the silver crystal is going to be investigated in the following.

### 2.2. Concentration Fluctuation Driving Silver Dynamic Crystallization

**Figure 3:**
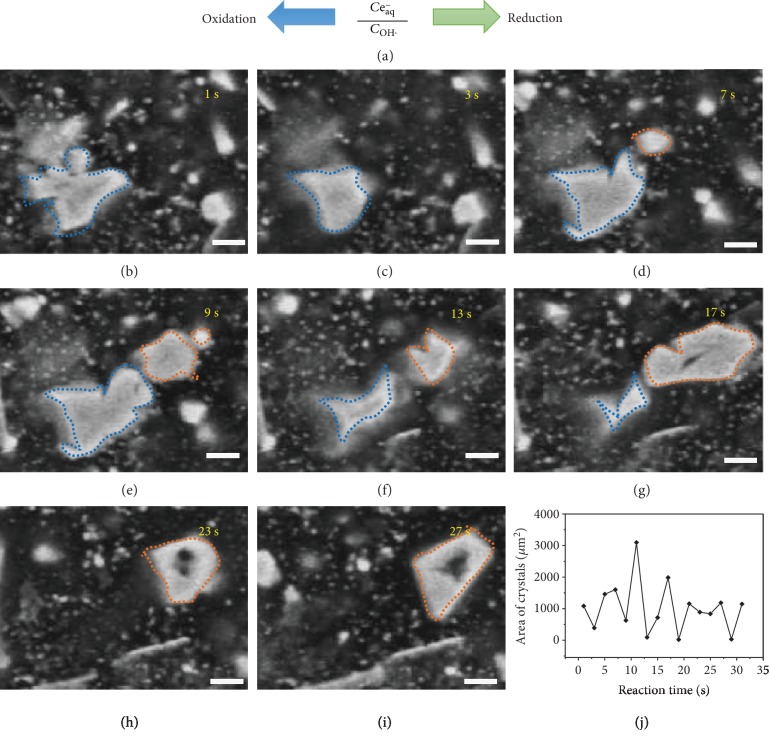
A reversible silver crystallization. (a) A radical concentration-dependent reaction. (b–i) The change of silver morphology with reaction time. (j) The quantization of the size of silver crystals. The scale bar is 5 *μ*m.

The silver crystallization in the liquid cell is recorded by a scanning electron microscope. When the electron beam hits the aqueous solution of the silver nitrate in the liquid cell, a reversible crystallization is observed, in which the formation of silver crystals is accompanied by the dissolution of crystals, as shown in the [Supplementary-material supplementary-material-1] (supporting information). Part of the video is decomposed to images, as shown in Figure 3. The white parts in Figures 3(b)–3(i) are silver crystals confirmed by EDS (energy-dispersive spectrometer) spectrum as shown in [Supplementary-material supplementary-material-1], while the black parts are solution. To track the formation of crystal particles, two particles are marked by a dotted line to circle their boundary, as shown in Figure 3. It is found that the shape and size of particles change with time. The growth and dissolution of particles coexists in the reactor. As far as our knowledge can reach, it is the first time to report the fluctuation of crystallization detected by electron microscopes. To quantify the size of crystals, the white area of crystals is measured by ImageJ software, as depicted in the experimental section. It is found that the white area fluctuates with irradiation time, as shown in Figure 3(j). In the combination of the finding of the fluctuation of radical concentration in the liquid cell, we propose that the fluctuation of radical concentration results in the reversible silver crystallization.

**Figure 4:**
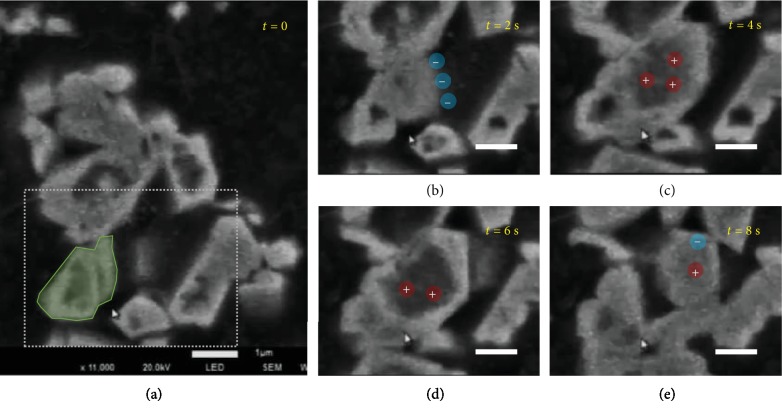
A focus on the dynamics of a silver crystal. (a) The green shadow pointing out the focused crystal. (b–d) A detailed time-dependent structure evolution of silver crystals in the liquid cell with an illustration of radical distribution around the crystal. The scale bar is 1 *μ*m.

To have a detailed view on the dynamic crystallization, one silver particle is focused, as shown in the green shadow of Figure 4(a). This particle is hollow inside and has an irregular shape. The flat surface outside indicates an orderly packing of silver atoms. After 2 seconds, the particle grows by extending the outside surface to the bulk and at the same time by replenishing the hollow structures inside, as shown in Figure 4(b). The growth of the silver particle indicates a classical crystallization without noticeable particle aggregation. After 4 seconds, the outside surface is further extended and the center of the particle becomes hollow again, as shown in Figure 4(c). This change is attributed to the heterogeneous distribution of reductive hydrated electrons and oxidative radicals. The bulk solution is the source of silver ions. Hence, the growth towards the bulk solution is preponderant. The reductive hydrate electrons diffuse to the outside surface of the particle due to the reaction there which consumes hydrate electrons resulting in a lower concentration. Therefore, the oxidative radicals are rich in the center of the particle, which leads to the dissolution of the particle in the center, forming a hollow structure, as shown in Figure 4(c). A further reaction to 6 seconds leads to the formation of flat surfaces outside and the hollow structure inside, as shown in Figure 4(d). The formation of a hollow structure creates a space for the silver nitrate solution, which triggers the growth of crystal at the inside surface, leading to the replenishment or disappearance of the hollow structure, as shown in Figure 4(e). Therefore, whether the particle grows or dissolves is determined by the proportion of the redox agents while the growth direction is dependent on the silver ion distribution, which confirms the dominant role of the chemical distribution in the structural evolution of materials.

### 2.3. Regulation of Electron Dose Rate to Form Diverse Silver Structures

**Figure 5:**
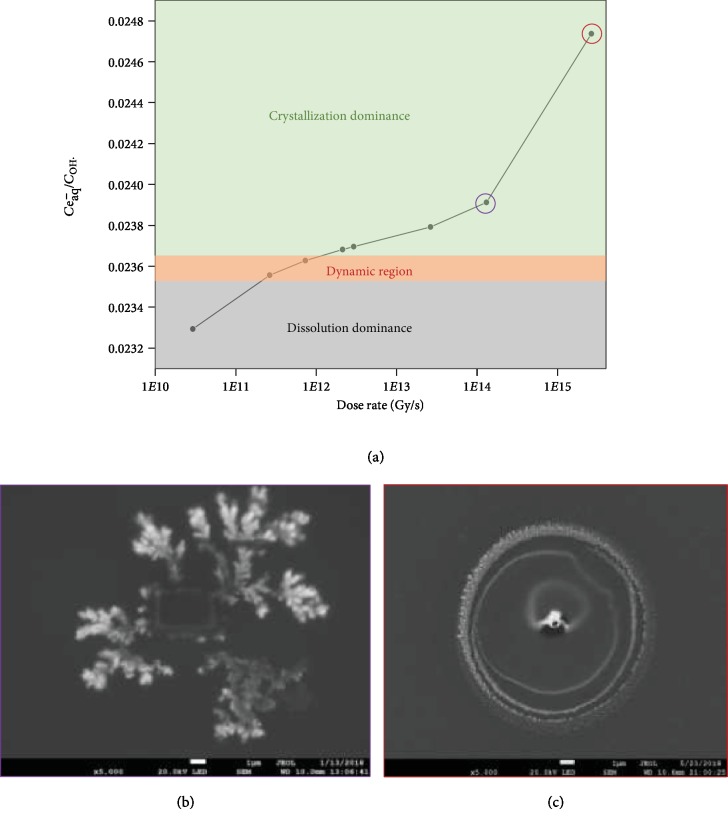
A regulation of reactions and silver morphologies. (a) The ratio of the concentrations of hydrated electron and hydroxyl radicals in the irradiation region at various electron dose rates. (b) The formation of dendritic structures and (c) multilayered structures outside the irradiation regions at the dose rates of 2.64 × 10^13^ Gy/s and 2.64 × 10^15^ Gy/s, respectively.

To further confirm the role of the chemical distribution in structural evolution, the *J* value is regulated by the electron dose rate which is dependent on the beam current, the acceleration voltage, and amplification. It is found that the *J* value increases with the electron dose rate, as shown in Figure 5(a), which agrees well with the reference [38]. The increase of hydrated electrons and hydroxyl radicals with the electron dose rate is shown in [Supplementary-material supplementary-material-1]. The concentration of hydrated electrons increased approximately 277 times while the concentration of hydroxyl radicals increases 248 times when the electron dose rate increases from 2.93 × 10^10^to 2.64 × 10^15^ Gy/s, which results in the increase of the *J* value. If the proposed dependence of the dynamic crystallization on the chemical distribution is correct, we expect the growth of crystals to predominate at a high dose rate while the dissolution of crystals is the majority at a low dose rate. When the electron dose rate is increased to 2.93 × 10^12^ Gy/s, the continuous growth of silver crystals is observed, as shown in the [Supplementary-material supplementary-material-1] (supporting information). When the sample is firstly irradiated at 2.93 × 10^12^ Gy/s, forming silver crystals, followed by a quick decrease of the electron dose rate to 2.93 × 10^10^ by reducing the magnification, a dissolution of crystals is observed, as shown in the [Supplementary-material supplementary-material-1] (supporting information).

When the electron dose rate is increased to 2.64 × 10^13^ Gy/s, silver dendritic structures are formed outside the irradiation region, as shown in Figure 5(b). The rectangle in the center is the area exposed to the electron beam. At a high dose rate, the generation of hydrated electrons and hydroxyl radicals is very fast, which leads to the formation of silver crystals which is limited by the transport of silver ions. The accumulation of hydrated electrons in the irradiation region drives their diffusion towards the bulk. The outside surface of the irradiation becomes the growth front of crystals. Owing to the high accumulation of hydrated electrons at the boundary, the silver crystallization take place at the outside surface of the boundary, in which the growth of crystals is limited by the diffusion of silver ions from the bulk. At a chemical diffusion limitation, silver dendritic structures are easily formed, which has been confirmed in the electrochemical experiments [29, 31]. The consumption of hydrated electrons makes hydroxyl radicals rich in the irradiation region, leading to the dissolution of silver crystals in the center. That is why there are no silver crystals in the center of the irradiation region. The formation of dendritic structures is also observed at the dose rate of 1.31 × 10^14^ Gy/s, as shown in the [Supplementary-material supplementary-material-1] (supporting information).

When the electron dose rate is increased to 2.64 × 10^15^ Gy/s, a white point is observed firstly. When the magnification is decreased, a circle with a diameter approximately 10 *μ*m is formed outside the irradiation region, as shown in Figure 5(c). The white circle is composed of silver nanoparticles. Several times of repeating zoom in and out lead to the formation of multilayered structures, as shown in Figure 5(c). The white point is caused by the charge accumulation, which is attributed to the formation of bubbles in the irradiated region. The bubble is close to the window of the liquid cell. A thin layer of liquid is formed between the solution and the silicon nitride window, which is highly prone to be charged due to poor conductivity of the bubble. Both hydrogen and oxygen are produced in the irradiation. Since the solubility of oxygen is higher than that of hydrogen in the solution, the hydrogen is the main composition of the bubble. Furthermore, the production of hydrogen increases with the dose rate, as shown in [Supplementary-material supplementary-material-1]. The hydroxyl radicals react with the hydrogen bubble, which slows down the diffusion of hydroxyl radicals from the bubble to the solution. In comparison, the diffusion of the hydrated electron from bubble to solution is preponderant. The difference of the diffusion rates of these two irradiated products leads to the formation of a multilayered circle, which agrees well with the formation mechanism of Turing patterns [39, 40]. Therefore, a regulation on the dose rate of the electron beam could change the chemical distribution in the reactor, leading to the formation of diverse silver structures.

## 3. Discussion

The dependence of chemical concentration on the structural evolution of crystals was investigated by employing an in situ scanning electron microscope. When the electron beam irradiated the liquid solution, various transient products including hydrated electrons and hydroxyl radicals, were generated. The reductive hydrate electrons and the oxidative hydroxyl radicals fluctuated in the cell due to the diffusion and reaction of radicals. This fluctuation led to a dynamic crystallization of silvers, as a result of the alternant dominance of the reduction and oxidation reactions in the cell. When the electron dose rate was increased, the formation of crystals became dominant while the dissolution predominated when the electron dose rates was decreased. More interesting results were observed at high dose rates. Silver dendrites were formed outside the irradiation region at the dose rate of 10^13^ Gy/s as a result of the diffusion limitation of silver ions. When the dose rate was increased to 10^15^ Gy/s, Turing circles were formed, which were attributed to the different diffusion rates of irradiated products at the bubble surface. Therefore, a regulation on the dose rate of an electron beam could change the radical distribution in the cell, leading to the formation of diverse structures. This study confirmed the dominant role of chemical distribution in the structural evolution of materials. A control of chemical concentration in the growth front of crystals may lead to the rational synthesis of material structures.

## 4. Materials and Methods

### 4.1. Materials and Reagents

Silver nitrate (AgNO_3_, 99.99% purity) was purchased from Sigma-Aldrich. Ethanol was provided by the Beijing Chemical Company. All chemicals were used without further purification. Deionized water from a Millipore (MQ) system with a resistivity higher than 18.2 M*Ω* cm were used in this study.

### 4.2. Preparation of a Liquid Cell to Be Loaded in a Scanning Electron Microscope

**Figure 6:**
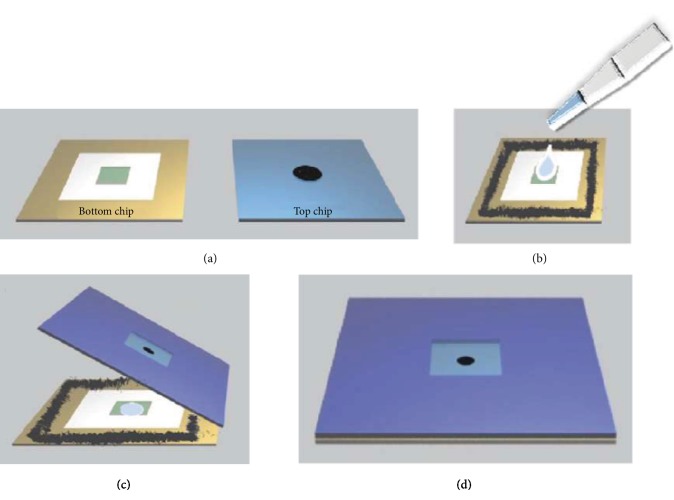
A preparation of liquid cell. (a–d) Schematic diagram of putting solution into a liquid cell for an in situ experiment in a scanning electron microscope.

The liquid cell from Zeptools (China, product model of MV-LSEM-1) was used in this study. The cell has a sandwiching structure with two 15 mm × 15 mm-square silicon chips layered together. The liquid layer is formed and sealed between chips. The center of the bottom chip contains a liquid reservoir with a depth of 100 *μ*m and dimensions of 3 × 3 mm, as shown in Figure 6(a). The solution of silver nitrate was added dropwise to the liquid reservoir by a micropipette, as shown in Figure 6(b). The top chip has an opening etched from the center with the size of 0.25 × 0.25 mm. An amorphous silicon nitride membrane with the thickness of 100 nm spans the opening to form the transparent window, which is a window transparent to the electron beam but capable of withstanding the pressure difference between the inside of the cell and the vacuum of the electron microscope. The epoxy resin glue was spread evenly on the edge of the bottom chip, followed by placing the top chip to seal the liquid film. The chips were aligned in one side and pressed to another side to avoid the formation of bubbles in the liquid, as shown in Figure 6(c). After the liquid cell was kept at room temperature (25°C) for 12 h, the leakage detection was carried out. If the window remains intact at the detecting pressure of 9.6 × 10^−5^ Pa, the liquid cell is ready to be placed in the scanning electron microscope for in situ experiments, as shown in Figure 6(d).

### 4.3. Quantification of the Radicals in the Liquid Cell

It is impossible to directly measure the transient radicals induced by the electron beam in the liquid cell owing to the short life of these radicals and the limitation of the microscope. The reactions induced by the irradiation are very complex [41]. The main products involved in the silver crystallization are the reductive and oxidative radicals. We employed a kinetic model [38] to quantify these radicals. The concentration *C*_*i*_(*x*, *t*) of the species *i* is described by the reaction-diffusion equation:

(1) $$\begin{array}{c} \frac{\partial C_{\text{i}}}{\partial t}=D_{i}\nabla^{2}C_{i}-\underset{j}{\sum }k_{ij}C_{i}C_{j}+\underset{j,k\neq i}{\sum }k_{ij}C_{i}C_{j}+R_{i}. \end{array}$$

The first term of equation (2) on the right side is the diffusion term. *D*_*i*_ is the diffusion coefficient of the product *i*. The second and third terms represent the destruction term and the production term of product *i* at the occurrence of relevant chemical reactions, respectively. *k*_*ij*_ is the corresponding chemical reaction rate. The last term *R*_*i*_ is the volumetric production rate of species *i* due to the irradiation in the region by electron beam. *R*_*i*_ is calculated by

(2) $$\begin{array}{c} R_{i}=\frac{\rho \Psi G_{i}}{F}\left(\text{M}/\text{s}\right). \end{array}$$

*G*_*i*_ represents the number of species *i* generated by the irradiation of unit energy. It is nonzero for the primary product in the irradiated region. *ρ* means the density of the liquid. *F* is the Faraday constant. Ψ represents the electron dose rate, which is calculated by

(3) $$\begin{array}{c} \Psi =\frac{S\cdot 10^{5}l}{\pi a^{2}}\left(\text{Gy}/\text{s}\right). \end{array}$$

*S* refers to the stopping power of the electron beam at different accelerating voltages. *l* is the beam size. *a* is the radius of the beam spot.

The concentration evolution of the irradiated product is obtained through running the MATLAB codes [25]. In the case when the liquid medium is irradiated by a beam point and the species diffuse in the irradiated region, the evolution of radical concentration is calculated by solving the diffusion-reaction equation via the component transport and finite chemical reaction modules in the ANSYS Fluent software.

### 4.4. In Situ Observing Silver Crystallization in the Liquid Cell

Filed emission scanning electron microscope (JSM 7800 Prime, JEOL) equipped with a camera (Epiphan DVI2USB3.0) was employed to conduct the in situ experiments. The beam current was quantified by the following equation.

(4) $$\begin{array}{c} \overset{\cdot }{d}=\frac{i_{e}}{eA}. \end{array}$$

Here, $\dot{d}$ is the electron dose rate (electrons/Å^2^s), *i*_*e*_ is the beam current (C/s), *e* is the elementary charge (C/electron), and *A* is the area of the scanning region (Å^2^) determined by the magnification. The electron dose per scan was calculated by multiplying $\dot{d}$ by the pixel dwell time and the number of pixels in the image. The electron dose rate ranges from 10^10^ to 10^15^ Gy/s in this study. Since the electron beam triggers chemical reactions at a very high rate (<1 *μ*s), the beam was focused on the edge of the chip firstly followed by moving the beam to the silicon nitride window, inducing reactions. The accelerating voltage was 20 kV and the probe current was 3 × 10^−10^ A. The freezing time was set to 0.2 s, and the working distance of the probe was kept at 10 mm. During the irradiation, the radicals formed in the liquid cell reacted with silver ions, forming silver crystals. The formation process of crystals was recorded by the camera inside of the microscope. The pixel of each image in the video is 1280 × 960. The growth of crystals was recorded by the Epiphan Capture Tool (DVI2USB 3.0) at a frame rate of fifteen frames per second. Still images were captured from the video by PotPlayer and analyzed by ImageJ software. The images were thresholded, and the pixel count areas were measured as a function of time. The size of silver particles in each image was quantified by calculating the area of crystals in the region exposed to the beam.

## Supplementary Materials

Supplementary Materials Figure S1: the EDS spectrum of solid particles formed in the liquid cell during the irradiation. Figure S2: the dependence of the concentrations of the hydrated electron. Figure S3: the concentration of hydrogen at various electron dose rates. Movie S1: a movie of the dynamic crystallization process at the electron dose rate of 7.33 × 10^11^ Gy/s. Movie S2: a movie of the formation of silver nanoparticles at the electron dose rate of 2.93 × 10^12^ Gy/s. Movie S3: a movie of the dissolution of silver nanoparticles at the electron dose rate of 2.93 × 10^10^ Gy/s. Movie S4: a movie of the formation of silver dendritic structures at the electron dose rate of 1.31 × 10^14^ Gy/s.

## References

[1] Liu Z., Chen D., Zhang J. (2018). Self-stabilized precipitation polymerization and its application. *Research*.

[2] Von Euw S., Zhang Q., Manichev V. (2017). Biological control of aragonite formation in stony corals. *Science*.

[3] Zhang X., He Y., Sushko M. L. (2017). Direction-specific van der Waals attraction between rutile TiO_2_ nanocrystals. *Science*.

[4] Chen C., Chen Y., Zhu S. (2018). Catalyst-free in situ carbon nanotube growth in confined space via high temperature gradient. *Research*.

[5] De Yoreo J. J., Gilbert P. U. P. A., Sommerdijk N. A. J. M. (2015). Crystallization by particle attachment in synthetic, biogenic, and geologic environments. *Science*.

[6] Widener (2013). Materials genome initiative. *Chemical and Engineering News*.

[7] Yu K., Xu T., Wu X. (2019). *In situ* observation of crystalline silicon growth from SiO_2_ at atomic scale. *Research*.

[8] Li J., Huang W., Chen J., Ge W., Hou C. (2018). Mesoscience based on the EMMS principle of compromise in competition. *Chemical Engineering Journal*.

[9] Yao Q., Chen T., Yuan X., Xie J. (2018). Toward total synthesis of thiolate-protected metal nanoclusters. *Accounts of Chemical Research*.

[10] Li Y., Zaluzhna O., Xu B., Gao Y., Modest J. M., Tong Y. Y. J. (2011). Mechanistic insights into the Brust-Schiffrin two-phase synthesis of organo-chalcogenate-protected metal nanoparticles. *Journal of the American Chemical Society*.

[11] Yao Q., Yuan X., Fung V. (2017). Understanding seed-mediated growth of gold nanoclusters at molecular level. *Nature Communications*.

[12] Cao Y., Guo J., Shi R. (2018). Evolution of thiolate-stabilized Ag nanoclusters from Ag-thiolate cluster intermediates. *Nature Communications*.

[13] Hu Q., Nielsen M. H., Freeman C. L. (2012). The thermodynamics of calcite nucleation at organic interfaces: classical vs. non-classical pathways. *Faraday Discussions*.

[14] Bots P., Benning L. G., Rodriguez-Blanco J. D., Roncal-Herrero T., Shaw S. (2012). Mechanistic insights into the crystallization of amorphous calcium carbonate (ACC). *Crystal Growth & Design*.

[15] Jiang J., Xu T., Lu J., Sun L., Ni Z. (2019). Defect engineering in 2D materials: precise manipulation and improved functionalities. *Research*.

[16] De Yoreo J. J. (2016). *In-situ* liquid phase TEM observations of nucleation and growth processes. *Progress in Crystal Growth and Characterization of Materials*.

[17] Nielsen M. H., Li D., Zhang H. (2014). Investigating processes of nanocrystal formation and transformation via liquid cell TEM. *Microscopy and Microanalysis*.

[18] Zhu G., Reiner H., Cölfen H., de Yoreo J. J. (2019). Addressing some of the technical challenges associated with liquid phase S/TEM studies of particle nucleation, growth and assembly. *Micron*.

[19] Liu L., Zhang S., Bowden M. E., Chaudhuri J., Yoreo J. J. D. (2018). In situ TEM and AFM investigation of morphological controls during the growth of single crystal BaWO_4_. *Crystal Growth & Design*.

[20] Park J., Elmlund H., Ercius P. (2015). 3D structure of individual nanocrystals in solution by electron microscopy. *Science*.

[21] Heo J., Dumett Torres D., Banerjee P., Jain P. K. (2019). In-situ electron microscopy mapping of an order-disorder transition in a superionic conductor. *Nature Communications*.

[22] Fu X., Chen B., Tang J., Hassan M. T., Zewail A. H. (2017). Imaging rotational dynamics of nanoparticles in liquid by 4D electron microscopy. *Science*.

[23] Liu Y., Lin X. M., Sun Y., Rajh T. (2013). In situ visualization of self-assembly of charged gold nanoparticles. *Journal of the American Chemical Society*.

[24] Shi Y., Yu Y., Liang Y., du Y., Zhang B. (2019). In situ electrochemical conversion of an ultrathin tannin nickel iron complex film as an efficient oxygen evolution reaction electrocatalyst. *Angewandte Chemie*.

[25] Grogan J. M., Schneider N. M., Ross F. M., Bau H. H. (2014). Bubble and pattern formation in liquid induced by an electron beam. *Nano Letters*.

[26] Turing A. M. (1953). The chemical basis of morphogenesis. *Philosophical Transactions of the Royal Society (Part B)*.

[27] Grzybowski B. A. (2009). *Chemistry in Motion: Reaction-Diffusion Systems for Micro- and Nanotechnology*.

[28] Tan Z., Chen S., Peng X., Zhang L., Gao C. (2018). Polyamide membranes with nanoscale Turing structures for water purification. *Science*.

[29] Liu W., Wang K., Zhou Y., Guan X., Che P., Han Y. (2019). Rational synthesis of silver nanowires at an electrode interface by diffusion limitation. *CrystEngComm*.

[30] Liu J., Yang T., Li C., Dai J., Han Y. (2015). Reversibly switching silver hierarchical structures via reaction kinetics. *Scientific Reports*.

[31] Yang T., Liu J., Dai J., Han Y. (2017). Shaping particles by chemical diffusion and reaction. *CrystEngComm*.

[32] Wang H., Han Y., Li J. (2013). Dominant role of compromise between diffusion and reaction in the formation of snow-shaped vaterite. *Crystal Growth & Design*.

[33] Park J., Zheng H., Lee W. C., Geissler P. L., Rabani E., Alivisatos A. P. (2012). Direct observation of nanoparticle superlattice formation by using liquid cell transmission electron microscopy. *ACS Nano*.

[34] Welch D. A., Woehl T. J., Park C., Faller R., Evans J. E., Browning N. D. (2016). Understanding the role of solvation forces on the preferential attachment of nanoparticles in liquid. *ACS Nano*.

[35] Noh K. W., Liu Y., Sun L., Dillon S. J. (2012). Challenges associated with in-situ TEM in environmental systems: the case of silver in aqueous solutions. *Ultramicroscopy*.

[36] Belloni J. (2006). Nucleation, growth and properties of nanoclusters studied by radiation chemistry: application to catalysis. *Catalysis Today*.

[37] Pastina B., LaVerne J. A. (2001). Effect of molecular hydrogen on hydrogen peroxide in water radiolysis. *The Journal of Physical Chemistry A*.

[38] Schneider N. M., Norton M. M., Mendel B. J., Grogan J. M., Ross F. M., Bau H. H. (2014). Electron-water interactions and implications for liquid cell electron microscopy. *The Journal of Physical Chemistry C*.

[39] Diambra L., Senthivel V. R., Menendez D. B., Isalan M. (2015). Cooperativity to increase Turing pattern space for synthetic biology. *ACS Synthetic Biology*.

[40] Mortazavi V., Nosonovsky M. (2011). Friction-induced pattern formation and Turing systems. *Langmuir*.

[41] Woehl T. J., Abellan P. (2017). Defining the radiation chemistry during liquid cell electron microscopy to enable visualization of nanomaterial growth and degradation dynamics. *Journal of Microscopy*.

