# Supplementary material for: In Situ Investigation of Dynamic Silver Crystallization Driven by Chemical Reaction and Diffusion
Source: Research (Wash D C). 2020 Feb 10;2020:4370817. doi: 10.34133/2020/4370817 (PMC7035454; doi:10.34133/2020/4370817)
Supplement: Supplementary Materials — Figure S1: the EDS spectrum of solid particles formed in the liquid cell during the irradiation. Figure S2: the dependence of the concentrations of the hydrated electron. Figure S3: the concentration of hydrogen at various electron dose rates. Movie S1: a movie of the dynamic crystallization process at the electron dose rate of 7.33 × 10^11^ Gy/s. Movie S2: a movie of the formation of silver nanoparticles at the electron dose rate of 2.93 × 10^12^ Gy/s. Movie S3: a movie of the dissolution of silver nanoparticles at the electron dose rate of 2.93 × 10^10^ Gy/s. Movie S4: a movie of the formation of silver dendritic structures at the electron dose rate of 1.31 × 10^14^ Gy/s. [file 4370817.f1.zip › Supporting Information-20191221.docx]

Supporting Information

**In-situ Investigation of Dynamic Silver Crystallization Driven by Chemicals Reaction and Diffusion**

Ting Liu^1,2,#^, Xiangyu Dou^1,3,#^, Yonghui Xu^1,3^, Yongjun Chen^2^, Yongsheng Han^1,3,*^

*1. State Key Laboratory of Multiphase Complex Systems,*

*Institute of Process Engineering, Chinese Academy of Sciences, 100190 Beijing, China*

*2. State Key Laboratory of Marine Resource Utilization in South China Sea,*

*Hainan University, 570228 Haikou, China*

*3. School of Chemical Engineering, University of Chinese Academy of Sciences, 100049 Beijing, China*

**Figure S1**


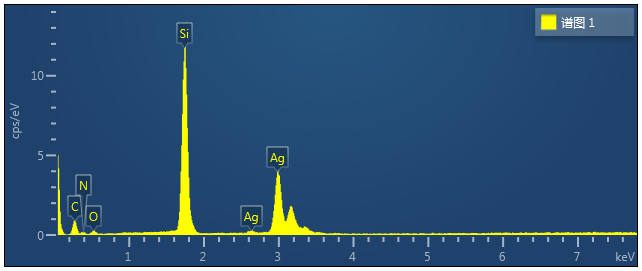


Fig. S1 shows the EDS (Energy dispersive spectrometer) spectrum of solid particles formed in the liquid cell during the irradiation, indicating that the main component of particles is silver. The high Si peak is attributed to the windows of silicon nitride membrane which is a window transparent to the electron beam but capable of withstanding the pressure difference between the inside of the cell and the vacuum of the electron microscope.

**Figure S2**

A

B

Fig. S2 shows the dependence of the concentrations of hydrated electron (A) and hydroxyl radical (B) on the electron dose rates, respectively.

**Figure S3**

Fig. S3 shows the concentration of hydrogen at various electron dose rates. With the increase of dose rates, the concentration of hydrogen increases largely. A sharp increase is found at the dose rate of 2.64×10^15^ Gy/s.
